# Immunization with M2e-Displaying T7 Bacteriophage Nanoparticles Protects against Influenza A Virus Challenge

**DOI:** 10.1371/journal.pone.0045765

**Published:** 2012-09-24

**Authors:** Hamidreza Hashemi, Somayeh Pouyanfard, Mojgan Bandehpour, Zahra Noroozbabaei, Bahram Kazemi, Xavier Saelens, Talat Mokhtari-Azad

**Affiliations:** 1 Department of Virology, Tehran University of Medical Sciences, Tehran, Iran; 2 Department of Virology, Tarbiat Modares University, Tehran, Iran; 3 Cellular and Molecular Biology Research Center, Shahid Beheshti University of Medical Sciences, Tehran, Iran; 4 Biotechnology Department, School of Medicine, Shahid Beheshti University of Medical Sciences, Tehran, Iran; 5 Department for Molecular Biomedical Research, VIB, Ghent, Belgium; 6 Department of Biomedical Molecular Biology, Ghent University, Ghent, Belgium; Johns Hopkins University - Bloomberg School of Public Health, United States of America

## Abstract

Considering the emergence of highly pathogenic influenza viruses and threat of worldwide pandemics, there is an urgent need to develop broadly-protective influenza vaccines. In this study, we demonstrate the potential of T7 bacteriophage-based nanoparticles with genetically fused ectodomain of influenza A virus M2 protein (T7-M2e) as a candidate universal flu vaccine. Immunization of mice with non-adjuvanted T7-M2e elicited M2e-specific serum antibody responses that were similar in magnitude to those elicited by M2e peptide administered in Freund’s adjuvant. Comparable IgG responses directed against T7 phage capsomers were induced following vaccination with wild type T7 or T7-M2e. T7-M2e immunization induced balanced amounts of IgG_1_ and IgG_2a_ antibodies and these antibodies specifically recognized native M2 on the surface of influenza A virus-infected mammalian cells. The frequency of IFN-γ-secreting T cells induced by T7-M2e nanoparticles was comparable to those elicited by M2e peptide emulsified in Freund’s adjuvant. Emulsification of T7-M2e nanoparticles in Freund’s adjuvant, however, induced a significantly stronger T cell response. Furthermore, T7-M2e-immunized mice were protected against lethal challenge with an H1N1 or an H3N2 virus, implying the induction of hetero-subtypic immunity in our mouse model. T7-M2e-immunized mice displayed considerable weight loss and had significantly reduced viral load in their lungs compared to controls. We conclude that display of M2e on the surface of T7 phage nanoparticles offers an efficient and economical opportunity to induce cross-protective M2e-based immunity against influenza A.

## Introduction

Influenza viruses are responsible for seasonal occurrences of influenza epidemics and infrequent, unpredictable worldwide pandemics. Each year 5–10% of the world population becomes infected with influenza viruses, resulting in considerable public health and economic burdens [1]. Currently licensed influenza vaccines rely mainly on the induction of neutralizing antibodies (Abs), which are directed mainly against the highly mutable influenza virus hemagglutinin (HA) envelope surface glycoprotein. Protection against influenza-associated illness by currently licensed vaccines is well-documented for most age-group. This protection relies on a close antigenic match between the HA present in the vaccine strains and that of the virus strains circulating in the population [2], [3], [4]. However, the antigenicity of HA changes repeatedly over time, a process known as antigenic drift, which is driven by escape mutants from the existing antibodies in the population [5], [6]. Therefore, the composition of seasonal influenza vaccines has to be updated almost every each year according to the results of global influenza surveillance performed by World Health Organization. This annual updating process represents quite a burden for vaccine manufacturers and in case of pandemic outbreaks, this strategy is futile for the control of the first wave on the pandemic. Influenza vaccines that are based on viral antigens that are more conserved within or even between influenza A virus subtypes, could offer a solution for this problem. One such a candidate “universal” influenza A vaccine has been developed pre-clinically as well as in phase I clinical studies [7], [8] and is based on the high sequence conservation exists in the ectodomain of the influenza virus channel protein M2 (M2e) among various subtypes of the virus. M2e consists of the 24 N-terminal amino acids of M2 [9]. Monoclonal antibodies against M2e have antiviral activity *in vitro* [10], although this is strain-dependent, and passive administration of such monoclonal antibodies can protect laboratory mice against influenza A virus-challenged mice [11]. Unlike most HA-specific Abs, anti-M2e Abs fail to neutralize the virions [10], [12], [13] and more likely exert their antiviral activity by binding to M2 proteins that are expressed in relatively large amounts on the membrane of infected host cells [9]. Although the exact mechanisms by which anti-M2e Abs protect against virus challenge have not yet been determined, both Ab-dependent cell-mediated cytotoxicity (ADCC) and complement-dependent cytotoxicity have been demonstrated to contribute to anti-M2e Ab-mediated immunity [14], [15]. Among IgG isotypes, IgG_2a_ and to a lesser extent IgG_2b_ have been shown to be the most important mediators of ADCC in mice [16], [17] and these are typically induced by Th_1_ cells that express cytokines such as IFN-γ upon activation [18]. In mice, M2e-specific IgG_2a_ (BALB/c) and IgG_2c_ (BL/6) offers stronger protection against influenza A virus challenged compared to IgG_1_ [15], [19].

M2e has a poor immunogenicity during a natural influenza A virus infection, presumably due to its limited expression on virions [20] and induces very low titers of M2e-specific antibodies in humans [21]. Hence, in recent years several researchers have been trying to improve the immunogenicity of the M2e peptide. It is believed that antigens can be rendered more immunogenic for B cells when presented in a highly repetitive and organized way, an approach that allows efficient cross-linking of B cell immunoglobulin receptors followed by increased signaling to B cells [22], [23], [24], [25]. For example, such an approach has been tested for M2e peptide by fusing it to a branched multiple antigenic peptide platform [26], [27] or by displaying M2e on the surface of various recombinant virus-like particles (VLPs) derived from hepatitis B virus core antigen, Papillomavirus L1 capsid protein, Papaya mosaic virus, phage QB as a chemical or genetic fusion [28], [29], [30], [31], [32]. Another interesting approach to enhance M2e-immunogenicity was by fusing M2e to the TLR5 ligand flagellin [29].

T7 phage nanoparticles have been exploited previously for display of peptides and B cell epitopes such as Ep15 peptide of West Nile virus [33] and an immuno-dominant region of Hepatitis B virus surface antigen (HBsAg) for diagnostic or vaccination purposes [34]. In this study, we developed a universal influenza vaccine based on T7 phage nanoparticles displaying M2e (T7-M2e). For this, we generated a genetic fusion of M2e with the 10B capsid protein of the T7 phage. Such phages could be easily purified and after removal of the co-purified bacterial lipopolysaccharide (LPS) the immunogenicity of the T7-M2e nanoparticles was assessed in mice. Humoral immune responses against both M2e peptide and structural proteins of T7 carrier were induced and M2e-specific IgG antibody isotypes (IgG_1_ and IgG_2a_) were further characterized and quantified by peptide- and infected cell-based ELISA. Finally, we demonstrated *in vivo* protection of T7-M2e nanoparticles against a lethal infection with H1N1 or H3N2 influenza A virus in a mouse model.

## Materials and Methods

### Ethics Statement

All procedures used in this study were approved by the Institutional Ethical Committee and Research Advisory Committee of Tehran University of Medical Sciences (May 21, 2011; proposal code 240/785) based on the National Specific Ethical Guidelines for Biomedical Research issued by Ministry of Health and Medicinal Education (MOHME) of Iran issued in 2005.

### Primer and Peptide Synthesis

All primers used in sequencing and cloning steps were synthesized and desalted by Eurofins MWG, Germany. Peptides corresponding to influenza A virus M2e (SSLLTEVETPIRNEWGCRCNGSSD) and a well-characterized *Plasmodium yoelii* H-2K^d^-restricted control peptide (SYVPSAEQI) [35], [36] were synthesized and HPLC purified (>98% purity) by Genscript (USA). Two potential overlapping M2e CTL epitopes (P_3–11_: LLTEVETPI ) and (P_7–15_: VETPIRNEW) were predicted *in silico* and similarly synthesized and purified. Peptides were provided as lyophilized preparations and reconstituted in sterile deionized water and stored at −20°C before use.

### Cloning of M2e in T7Select 415-1b Genomic Arms and Generation of T7-M2e Phages

**Figure 1:**
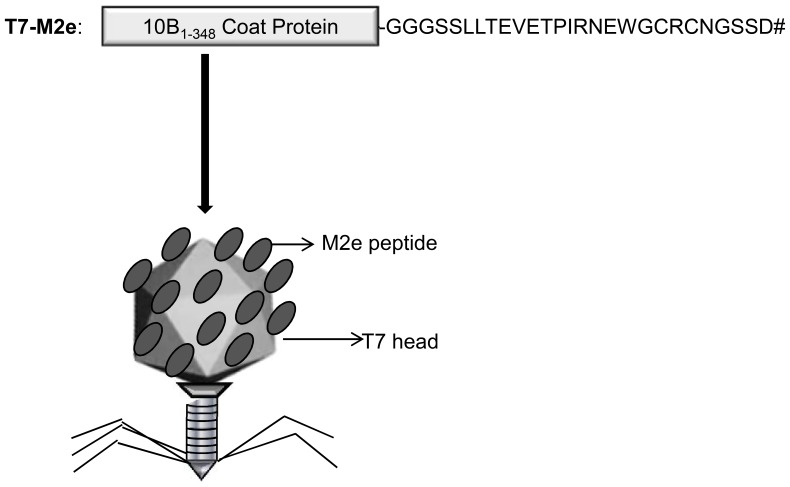
Schematic diagram of the 10B-M2e chimeric capsid protein displayed on the T7-M2e phage nanoparticles. M2e peptide with a GGGS linker is fused to C-terminus of 10B capsid protein after amino acid residue 348 of 10B coat protein. TAA stop codon (#) was inserted downstream of the M2e sequence to avoid any C-terminus extension of the M2e peptide.

The oligonucleotide encoding M2e peptide with a glycine-glycine-glycine-serine (GGGS) linker was codon optimized according to the codon usage table of *Escherichia coli* strain B in Codon Usage Database (http://www.kazusa.or.jp/codon/) using Eurofins MWG online software, GENEius. The synthetic M2e insert was first cloned into pCDNA3.1, which served as a template for amplification by a high-fidelity PCR using pfu DNA polymerase (Fermentas), pcDNA3.1-M2e template and the flanking primers (Forward: 5′-TAGCGGTTTGACTCACGG-3′) and (Reverse: 5′-ATGCCTGCTATTGTCTTCC-3′). The PCR product was digested with EcoRI and HindIII restriction enzymes and the M2e-endoding 103 bp insert was purified. The T7Select415-1b cloning kit containing the T7415-1bEcoRI/HindIII double-digested and dephosphorylated T7 phage genomic arms (Novagen, Germany) was used to display M2e peptide on T7 phage head as a fusion to the C-terminus of 10B capsid protein (Fig. 1). The T7 capsid shell contains 415 copies of 10B capsid protein, thus M2e is displayed on the resulting T7 nanoparticles with a high copy number. The ligation reaction was performed with the T4 DNA Ligase (Fermentas) and overnight incubation at 16°C. Four microliters of the ligation reaction was mixed with 25 µl of T7 phage packaging extract and incubated for 2 h at room temperature. Full-length wild type T7 phage genomic DNA was also packaged and used as control phage nanoparticles (T7-wt) throughout the study. The reactions were stopped by adding 270 µl of sterile Luria-Bertani (LB) broth and the packaging efficiency was determined by titration by plaque assay using *E. coli* BL21 as a host according to the technique described by Adams [37]. The plaques were counted and the phage titers expressed as PFU/ml.

### Immunoscreening of Recombinant T7-M2e Phage Plaques

Immunoscreening of T7 plaques was performed as described by Jinag *et al.* for T4 phage with some modifications [38]. In brief, T7 bacteriophage plaques resulting from T7-M2e or T7-wt packaging reactions were transferred to nitrocellulose membranes for 30 min at 4°C, air-dried for 15 min and blocked with skim milk (5% in TBS) for 1 hour. The membranes were washed once in TBS containing 0.05% Tween-20 and incubated with 1/1000 dilution of anti-M2e monoclonal antibody 14C2 (Abcam) in the same buffer for 2 hours at RT. The bound antibodies were detected by an alkaline phosphatase (AP)-conjugated goat anti-mouse IgG (Abcam) and subsequent addition of a chromogen mixture of 5-Bromo-4-Chloro-3 Indolylphosphate p-Toluidine salt (BCIP) and Nitro Blue Tetrazolium Chloride (NBT). When the intensities of phosphatase catalyzed plaque staining were judged as strong enough, the membranes were rinsed with deionized water and dried at room temperature. Positive M2e-dislaying plaques were picked by aligning the membrane with plates. PCR on plaque-derived material was performed according to manufacturers’ instruction. Briefly, a portion of the top agarose of an individual plaque of interest was scraped and dispersed in a tube containing 100 µl of 10 mM EDTA, pH 8.0. The tube was heated for 10 min at 65°C and clarified by centrifugation at 14000 g for 3 min. PCR reaction was performed on 2 µl of the clarified sample using Taq DNA polymerase (Fermentas) and T7 specific primers (Up primer: 5′-GGAGCTGTCGTATTCCAGTC-3′ and Down primer: 5′-AACCCCTCAAGACCCGTTTA-3′).

### Large-scale Amplification and Purification of T7-M2e Phage Nanoparticles

A volume of 1000 ml M9LB medium (Luria-Bertani broth supplemented with 50 ml 20X M9 salts, 20 ml 20% glucose and1 ml 1 M MgSO_4_ per liter) was inoculated with 10 µl of a saturated culture of BL21 and incubated overnight at 30°C to reach a final OD_600 nm_ = 0.8–1. The number of cells in the culture medium was determined and bacteria were infected with the phage nanoparticles at a multiplicity of infection (MOI) of 0.001. The culture was incubated by vigorous shaking at 37°C until complete lysis of cells was observed, which typically required 3–6 hours. To degrade bacterial DNA and RNA released during lysis, DNase I and RNase A (Roche, USA) were added 20 min before harvesting the phages from the culture medium. After addition of solid NaCl (1 M), the suspension was centrifuged for 25 min at 4000 g to separate the bacterial debris from the T7 phage nanoparticles. The resulting supernatant was stored overnight at 4°C after addition of 10% polyethylene glycol (PEG 6000; Sigma). The mixture was centrifuged for 15 min at 11000 rpm and 4°C. The supernatant was decanted and the pellet containing the T7 phages was resuspended in a buffer solution of 1 M NaCl, 10 mM Tris–HCl pH 8 and 1 mM EDTA. The PEG was removed by two consecutive centrifugations for 15 min at 10,000 rpm, followed by extraction with an equal volume of chloroform to remove residual PEG and bacterial debris. After centrifugation at 3000 g and 4°C for 10 min, the upper aqueous phase containing T7 phage nanoparticles was transferred to a new tube. The phage nanoparticles were further purified by ultracentrifugation on a four-step gradient of cesium chloride (CsCl) in Tris-HCl buffer according to manufacturer’s instructions (40000 rpm, 35 min at 20°C). The band containing phage particles was isolated from the gradient and residual CsCl was removed by three consecutive exchanges with pyrogen-free PBS and concentrated in a centrifugal ultrafiltration device (Sartorius) with 100 KDa molecular weight cut-off (MWCO). The purified T7 nanoparticles were sterilized using a pyrogen-free 0.2 µm pore size cellulose acetate filter (Millipore) and quantified by plaque assay as described.

### Endotoxin Removal from T7-M2e Phage Nanoparticles

The bacterial endotoxin (LPS) concentration in all T7 nanoparticle preparations was determined in triplicate using a sensitive colorimetric Limulus Amebocyte Lysate (LAL) QCL-1000® kit (Lonza, USA) according to the manufacturer’s instructions. LPS was removed from T7 phage nanoparticles based on a method for removal of endotoxin from protein solutions by phase separation using Triton X-114 as described by Aida *et al*. [39] and modified by Hashemi *et al*. (manuscript submitted for publication).

### SDS-PAGE and Western Blotting

**Figure 2:**
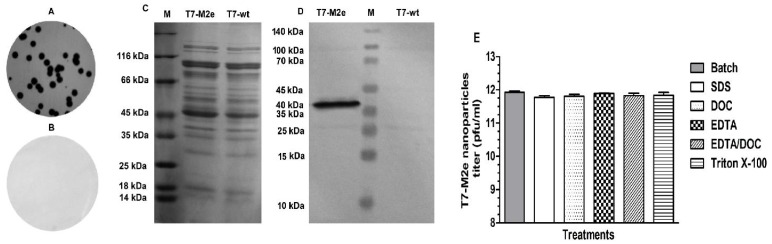
Plaque lift assay on T7-M2e and T7-wt plaques. Plaques developed after in vitro packaging reaction were transferred onto nitrocellulose membranes. After incubation with 14C2 Ab followed by anti-mouse polyclonal conjugate and development by BCIP/NBT chromogen, sharp spots were developed immediately (2–3 min) indicating accessibility and immunoreactivity of M2e peptide when displayed on the surface of T7 nanoparticles (A). No spots are observed on T7-wt phage plaque lift (B). Following amplification and purification of T7 phage nanoparticles, capsid proteins were separated by SDS-PAGE on 12% polyacrylamide gel (C) and 10B-M2e fusion protein (40 kDa MW) was detected by Western blotting on nitrocellulose membrane using 14C2 anti-M2e monoclonal antibody (D). As shown, no bands were detected on the T7-wt lane indicating no reactivity of 14C2 mAb with capsid proteins. M lane: protein marker. (E) Assessment of T7-M2e stability against various harsh conditions. There was no significant change in the T7-M2e phage titer after incubation for 60 min at 37°C. Results are reported as PFU/ml.

SDS-PAGE was performed according to the method described by Laemmli [40]. Briefly, purified T7-M2e and T7-wt nanoparticles (as negative control) were mixed with 2x-sample buffer [(62.5 mM Tris-HCl (pH 6.8), 25% glycerol, 2% SDS, 0.01% bromophenol blue and 200 mM dithiothreitol (DTT)] and boiled for 10 min. Western blotting was performed according to the method of Towbin [41]. The protein samples were electrophoresed on a 12% polyacrylamide gel and stained with Coomassie brilliant blue (Fig. 2C). For Western blotting, proteins were transferred onto nitrocellulose membranes (Sigma) using a semi-dry blotting device (APELEX, France) for 2 h. Membranes were blocked with 5% skim milk in TBS overnight at 4°C. Anti-M2e monoclonal antibody 14C2 (Abcam) was added in TBS plus 0.1% BSA at final concentration of 1 µg/ml followed by extensive washing with TBS plus 0.05% Tween-20. Next, the membrane was incubated with AP-conjugated goat anti-mouse IgG polyclonal antibody (Abcam) for 1 hr and the protein bands were visualized by adding a mixture of BCIP/NBT (Novagen) (Fig. 2D).

### Immunization of Mice

Immunogenicity studies were performed in 6–8 weeks old female BALB/c mice (Pasteur Institute, Iran) and vaccine preparations were injected subcutaneously. T7 and T7-M2e phage nanoparticles with or without Freund’s adjuvant were injected at 10^9^ pfu per mouse. As a positive control 100 µg of the synthetic M2e peptide adjuvanted with Freund’s adjuvant was used as a vaccine. In addition, T7 nanoparticles mixed with 100 µg of the free synthetic M2e peptide (T7-wt+M2e group) were administered to assess whether multivalent display of M2e on the surface of T7 capsids is critical for eliciting an immune response against this peptide. Separate groups of mice received PBS or Freund’s adjuvant (CFA/IFA group) as negative controls. Mice were primed on day 0 and boosted twice at 2-week intervals. In the case of adjuvanted nanoparticles or M2e peptide, the antigen was administered in CFA (Sigma) on day 0 and in IFA (Sigma) on days 14 and 28. Mice were housed in an environment with 12-h light/dark cycles and received food and water *ad libitum*.

### Evaluation of M2e-specific and Anti-T7 Carrier IgG Antibodies by ELISA

Mice were bled through the retro-orbital vein 20 days after the third immunization to determine pre-challenge IgG titers. Blood samples were left to clot at RT for 1 h followed by at least 5 h at 4°C for contraction of clots. The sera were collected by centrifugation for 10 min at 1200 rpm and stored at −20°C until analysis. The titers of total IgG and isotypes (IgG_1_ and IgG_2a_) in serum directed against the M2e peptide and T7 nanoparticles (anti-carrier Abs) were determined by ELISA. Ninety six-well high binding ELISA microplates (COSTAR, USA) were coated with 10^9^ T7-wt phage nanoparticles in Tris-NaCl resuspension buffer (PH 8) or M2e peptide (10 µg/ml) in carbonate-bicarbonate buffer (100 mM, PH 9.6) for 1 hour at RT and then overnight at 4°C. Plates were blocked with 5% skim milk in PBS for 1 hour at 37°C and washed once with PBS containing 0.05% Tween-20 (PBST) buffer. Sera were serially diluted in PBS plus 0.1% BSA and added to wells. After incubation at 37°C for 2 h and performing washing steps, AP-conjugated goat polyclonal anti-mouse IgG (1/20,000 dilution), anti-mouse IgG_1_ (1/5000 dilution) or IgG_2a_ (1/5000 dilution) was added. A yellow color was developed after addition of SIGMA-FAST para-nitro phenyl phosphate (pNPP) substrate in Tris buffer which was stopped by addition of 50 µl of 3N NaOH. Absorbance of the plates was read at 405 nm using a microplate ELISA reader (TECAN). All serum samples from individual mice were assessed in triplicate.

### Binding Capacity of Anti-sera to M2 Expressed on Influenza Virus-infected Cells

Immune sera were assessed for specific binding to MDCK cells infected with PR8 virus as described previously [21]. Briefly, MDCK cells were grown to near confluence in 96-well plates in DMEM containing 10% FBS at 37°C and 5% CO2. The cells were infected by 1 multiplicity of infection (MOI) of PR8 virus in DMEM without serum or incubated with medium alone as uninfected control. After 1 hour incubation at 37°C, DMEM medium (without serum) was added to each well and plates were incubated for 12 hours at 37°C. Next, plates were washed thoroughly in cold complete medium, fixed with 10% formalin at room temperature for 10 min, washed with PBS and blocked with 200 µl/well of 1% BSA in PBS for 1 hour. Sera from mice in each group were pooled, two-fold diluted and added to wells in triplicate. Serial dilutions of mice sera in PBS containing 0.1% BSA were added to the wells and incubated for 90 min at room temperature. After washing with PBS, HRP-conjugated goat anti-mouse IgG (Abcam) was added and incubated for 1 hour at room temperature followed by detection with Tetramethylbenzidine (TMB) substrate. The same treatments were performed on non-infected MDCK cells to determine background immune-reactivity of the anti-sera. The reaction was stopped by adding 50 µl of 1 M H_2_SO_4_ and OD_450 nm_ was measured on a microplate spectrophotometer (TECAN).

### Isolation of Spleen Cells

The day before challenge with influenza viruses (20 days after the second booster) 5 mice from each group were sacrificed by cervical dislocation. Spleens were aseptically removed and splenocytes were harvested after homogenization and removal of debris by filtration through a 70 µm nylon cell strainer (BD). Red blood cells (RBCs) were lysed using a Tris-ammonium chloride buffer (NH_4_Cl 0.16 M, Tris-HCl 0.17 M) and splenocytes were washed several times with PBS. Finally splenocytes were resuspended in complete RPMI-1640 medium containing 10% fetal bovine serum (FBS), 2 mM L-glutamine, 1 mM sodium pyruvate, 100 IU/ml penicillin, 100 µg/ml streptomycin and HEPES buffer. Cell viability was determined by trypan blue dye (0.4% w/v) exclusion.

### Measurement of M2e-specific T Cell Responses by Interferon-γ (IFN-γ) ELISPOT Assay

Specific T-cell responses to M2 peptide were assessed *ex vivo* by a mouse IFN-γ Enzyme-linked Immunosorbent Spot (ELISPOT) Assay kit (eBioscience). Ninety six-well Multiscreen IP Plates (Millipore) were coated with 100 µl of assay diluent containing anti–IFN-γ monoclonal Ab and incubated overnight at 4°C. The plates were washed and then blocked with RPMI-1640 medium containing 10% fetal bovine serum (FBS) for 60–90 min at room temperature. Splenocytes depleted of erythrocytes were added to wells (300,000 cells/well in 100 µl) in triplicate and stimulated with M2e Peptide (10 µg/ml). Furthermore, we performed an *in silico* analysis of M2e peptide to predict potential H-2K^d^-restricted CTL epitopes. Two overlapping 9-mer peptides with the highest MHC binding affinity, proteasome cleavage and TAP transport scores were selected (P_3–11_: LLTEVETPI and P_7–15_: VETPIRNEW) and added to the wells in the same concentrations as full-length M2e. The mitogen phytohemagglutinin (PHA) was added to positive control wells at final concentration of 4 µg/ml and Plasmodium yoelii H-2K^d^ peptide (as irrelevant peptide: SYVPSAEQI) was used as a negative control (10 µg/ml). After incubation for 36 h at 37°C and 5% CO2, the bound IFN-γ was detected with a biotinylated monoclonal antibody. Spots were developed on the membranes using HRP-conjugated streptavidin and a mixture of aminoethylcarbazole (AEC) (Sigma) substrate and H_2_O_2_ in acetate buffer and counted under a dissection microscope (Nikon).

### Amplification and Titration of Influenza Viruses

The mouse-adapted influenza A viruses used for challenge studies were A/Puerto Rico/8/34 (PR8) and X47 virus (a reassortant H3N2 subtype virus) [42]. Viruses were amplified by inoculation into the allantoic cavity of 10-day-old embryonated chicken eggs. The eggs were incubated at 35°C for 3 days and then chilled at 4°C. The allantoic fluids were harvested and tested for presence of haemagglutinin (HA) activity by mixing 50 µl of supernatant with 50 µ1 of a 0.5% suspension of guinea pig RBCs. Madin-Darby Canine kidney cells (MDCK) were inoculated with serial log_10_ dilutions of the clarified allantoic fluids and the 50% tissue culture infectious dose (TCID_50_) was calculated after 72 hours. For challenge of mice, the viruses were quantified as 50% mouse lethal dose (MLD_50_) by the Reed-Muench method.

### Challenge of Mice with Influenza Viruses

Three weeks after the second booster injection, mice were anesthetized with a mixture of 10% ketamine (100 mg/kg) and 2% xylazine (10 mg/kg) and challenged by intranasal administration of 50 µl PBS containing 4 LD_50_ of mouse-adapted PR8 or X47 virus. Mortality and clinical symptoms including weight loss, ruffled fur, hunched posture, rapid breathing, inactivity and paralysis of posterior limb were monitored for 14 days after challenge.

### Determination of Viral Loads in Lungs

Five days after challenge, five mice from each group were sacrificed by cervical dislocation for titration of residual lung virus. Lungs were aseptically removed from mice, rinsed in sterile PBS and homogenized in 2 ml ice-cold PBS containing 100 IU/ml penicillin and 100 µg/ml streptomycin. Lung homogenates were clarified by centrifugation for 10 min at 2400 rpm to pellet cell debris and the supernatants stored at −20°C until assay. Semi-confluent MDCK cells in 96-well plates were infected with serial log_10_ dilutions of clarified lung samples in DMEM containing TPCK-treated trypsin. After incubation for 72 h at 37°C in 5% CO2, the culture supernatants were tested for the presence of influenza virus HA activity by mixing 50 µl of supernatant with 50 µl of a 0.5% suspension of guinea pig RBCs. The virus titer for each lung specimen was calculated by the Reed-Muench method and expressed as the 50% tissue culture infective dose (TCID_50_). The virus titers are represented by the mean ± SD of the virus titer per ml of the lung homogenates prepared from five mice in each group.

### Statistical Analysis

Antibody titers, body weight changes and lung residual virus titers were compared among different groups of mice by Student’s *t* test and one-way ANOVA followed by Turkey’s and Dunnett’s post-tests. Analysis of T cell data was performed using one-way ANOVA followed by Turkey’s test. Survival data were analyzed by Kaplan–Meier method and log-rank test was used to analyze differences among survival curves. Values of *p<0.05, **p<0.01, ***p<0.001 were considered statistically significant. All statistical analyses were performed using the GraphPad Prism 5 software (La Jolla, CA, USA).

## Results

### M2e is Efficiently Displayed on T7-phage Nanoparticles

A high titer of T7-M2e phage nanoparticles (10^11^ pfu/ml) was obtained by infection of *E. coli* BL21 culture at MOI = 0.01–0.001 after a few hours of incubation (3–5 h). Display of M2e in plaques derived from T7-M2e phages was demonstrated in a bacteriophage plaque lift assay using the M2e-specific monoclonal antibody 14C2 (Fig. 2A, B). T7 phages are known to be very stable in the environment [43]. To evaluate the stability of the M2e-modified T7 phages, we incubated T7-wt and T7-M2e phages in 1% SDS (a strong denaturing anionic detergent), 1% sodium deoxycholate (DOC) and 10 mM EDTA (a strong Ca^2+^ and Mg^2+^ ion chelator, which destabilizes many protein-protein interactions) and a combination of these agents for 1 h at 37°C. Such incubations did not compromise the integrity of T7 nanoparticles as assessed by plaque assay (Figure 2E). These results indicate that display of M2e peptide on all 415 monomers of 10B capsid protein did not interfere with the assembly of T7-M2e nanoparticles in infected bacteria. In addition, the purified T7-M2e phages retained their infectivity following incubation in harsh denaturing conditions, suggesting that banked T7-M2e phages would be a reliable inoculum source to produce M2e-vaccine [43].

### T7-M2e Phage Nanoparticles Elicit a Robust Anti-M2e and Anti-T7 Carrier IgG Antibody Response

**Figure 3:**
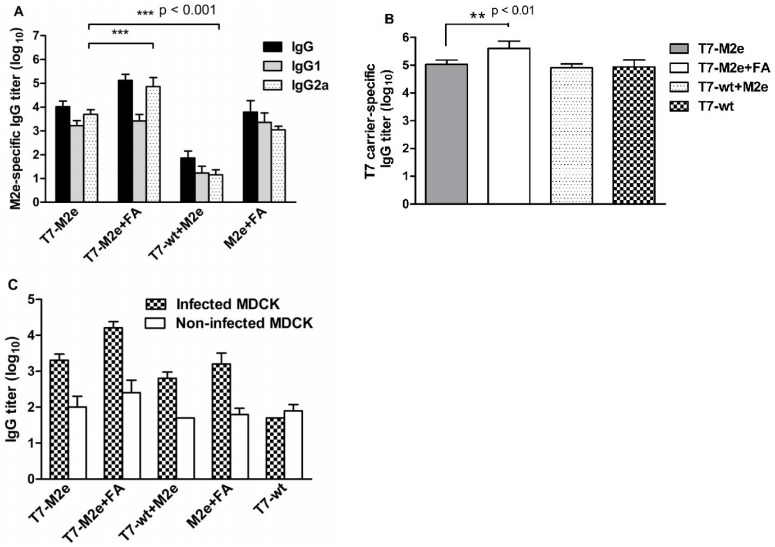
Antibody responses elicited by T7-M2e nanoparticles. (A) Total IgG, IgG_1_ and IgG_2a_ antibody titers against the influenza virus M2e peptide. Results are expressed as an antibody endpoint titer, where the O.D. value is 3-fold higher than the background value obtained with a 1∶50 dilution of pre-immune serum from BALB/c mice. Data represent the mean ±SD of five mice. Statistically significant differences are represented by *p<0.05, **p<0.01, and ***p<0.001. (B) Measurement of anti-T7 nanoparticle IgG antibodies by ELISA using T7-wt-coated microplates. Each value represents mean ± SD in each group of mice. (C) Binding capacity of antibodies in vaccinated mice sera to native M2 expressed on PR8 virus-infected MDCK cells. Results from PBS and CFA/IFA groups are not shown as there was no significant difference compared to T7-wt group. Details are described in Results.

To determine the immunogenicity of our T7-M2e based nanoparticles, we immunized BALB/c mice via the subcutaneous route with purified, endotoxin-free phages in the presence or absence of Freund’s adjuvant. In the absence of adjuvant, T7-M2e phage nanoparticles elicited a high titer of serum anti-M2e IgG antibody response as determined by M2e peptide ELISA (Fig. 3a). This suggests that T7 phage nanoparticles are highly immunogenic in mice even in the absence of additional adjuvants or contaminating bacterial LPS. Moreover, high levels of anti-T7 carrier IgG antibodies were induced against structural proteins of T7 nanoparticles as measured by serum ELISA performed on plates coated with T7-wt nanoparticles (Fig. 3b). Because we observed a high titer of IgG Abs against both T7 nanoparticle capsids and M2e peptide, we concluded that carrier-specific antibodies did not suppress the development of an M2e-specific IgG response in immunized mice. Isotypes of M2e-specific IgG in anti-sera were characterized by ELISA using IgG_1_- and IgG_2a_-specific secondary antibodies. As shown in figure 3, T7-M2e nanoparticles induced a balanced amount of both IgG_1_ and IgG_2a_ antibodies in mice. Immunization in the presence of CFA/IFA adjuvant to T7-M2e nanoparticles (T7-M2e+FA group) significantly increased both M2e-specific and anti-T7 carrier IgG antibodies compared to the non-adjuvanted setting (p<0.01). Moreover, anti-M2e IgG_2a_ antibody level was significantly higher in mice receiving T7-M2e nanoparticles formulated in Freund’s adjuvant compared to adjuvant-free immunization (p<0.001) indicating the induction of a Th_1_-dominant immune response, whereas no significant difference was observed in IgG_1_ titers. To assess this surmised M2e-specific Th_1_-response, we quantified the number of IFN-γ-secreting splenocytes by ELISPOT.

Sera from mice that had been immunized with a mixture of T7-wt nanoparticles and free M2e peptide (T7-wt+M2e group) contained significantly lower titers of anti-M2e antibodies compared to T7-M2e immunized mice, indicating the importance of multivalent arrangement of M2e peptide on the surface of T7 nanoparticles in eliciting a strong antibody response. This finding is in accordance with the results obtained by previous studies using Hepatitis B core, phage Qß or Papaya mosaic virus VLPs [22], [32], [44]. Control mice inoculated with wild-type T7 phage nanoparticles (T7-wt), PBS or CFA/IFA had no detectable anti-M2e Abs. As previously reported, M2e peptide injected with CFA/IFA (M2e+FA group) induced high titers of M2e-specific IgG_1_ and IgG_2a_ antibodies [45], [46] even though these were significantly lower than those induced by T7-M2e emulsified in CFA/IFA (p<0.001).

### Abs Elicited by T7-M2e Nanoparticles Efficiently Recognize Native Tetrameric M2 on Influenza Virus-infected Cells

Considering that we used a multivalent display approach for a linear format of the M2e peptide on the surface of icosahedral capsids of T7 phage nanoparticles, we investigated whether the elicited antibodies could also recognize native tetrameric M2 expressed on the surface of infected cells. To analyze such responses, we used an MDCK cell-based whole cell ELISA. As shown in Fig. 3c, sera from mice immunized with T7-M2e efficiently recognized native M2 expressed by PR8 virus-infected MDCK cells, whereas only background binding was observed with sera derived from mice that had received T7-wt, PBS or CFA/IFA (p<0.01). Similar to the results obtained with M2e peptide-coated ELISA plates, sera from mice administered with an emulsion of T7-M2e in CFA/IFA showed a higher binding to virus-infected MDCK cells although this difference was not statistically significant. Furthermore, antibodies from the T7-M2e+FA group bound more strongly to PR8 infected-MDCK cells compared to sera from mice that had been immunized with M2e peptide in CFA/IFA.

### Immunization with T7-M2e Phage Nanoparticles Induces M2e-specific T Cell Responses

**Figure 4:**
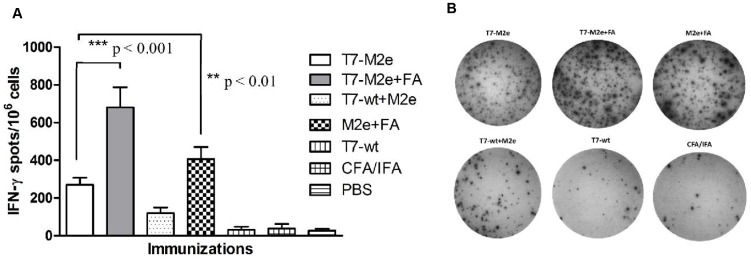
M2e-specific T cell responses were measured using IFN-γ ELISPOT. Splenocytes were isolated from immunized mice, pooled in each group and assayed for IFN-γ-producing cells by ELISPOT as described in Materials and Methods. (A) Data are represented as number of IFN-positive spot forming cells (SFCs) per 10^6^ pooled splenocytes in each group ± SD. All samples were tested in triplicate. (B) Frequency of IFN-γ spots in each group of mice.

To evaluate T cell-mediated immunity to M2e peptide, splenocytes from immunized mice were stimulated *in vitro* with synthetic M2e peptide and the numbers of IFN-γ-secreting cells were determined by ELISPOT. As shown in figure 4, the mice immunized with M2e peptide plus Freund’s adjuvant or T7-M2e nanoparticles (with or without adjuvant) showed a significantly higher frequency of M2e-specific IFN-γ-secreting cells compared to negative controls (T7-wt, CFA/IFA and PBS) (p<0.001) and those receiving a mixture of T7-wt and M2e peptide (p<0.01). Moreover, T cell responses provoked by T7-M2e nanoparticles were significantly greater than T7-wt nanoparticles combined with synthetic M2e peptide (p<0.01) which correlates with the higher anti-M2e IgG titers observed in sera from T7-M2e immunized mice (Fig. 3). Co-administration of Freund’s adjuvant with T7-M2e nanoparticles (T7-M2e+FA group) resulted in a significantly higher number of IFN-γ-producing cells compared to adjuvant-free immunizations or M2e peptide emulsified in Freund’s adjuvant (p<0.001). However, splenocytes from mice receiving M2e peptide emulsified in Freund’s adjuvant had a significantly greater frequency of IFN-γ-secreting T cells than those receiving T7-M2e nanoparticles without adjuvant (p<0.01). The number of spots in control mice immunized with T7-wt nanoparticles, CFA/IFA or PBS was low and similar to those obtained after *in vitro* stimulation with the irrelevant H-2k^d^ peptide. Moreover, no significant difference was observed between the number of IFN-γ spots from the splenocytes stimulated with the irrelevant control peptide and the *in silico* predicted CTL peptides, P_3–11_ and P_7–15_ (data not shown). The latter result indicates that the T7-M2e immunogen does not give rise to cross-presentation in our model, or that the predicted peptides are poor CTL epitopes in the H-2k^d^ background.

### T7-M2e Nanoparticles without Adjuvant Protect Mice Against both H1N1 (PR8) and H3N2 (X47) Virus Challenge

**Figure 5:**
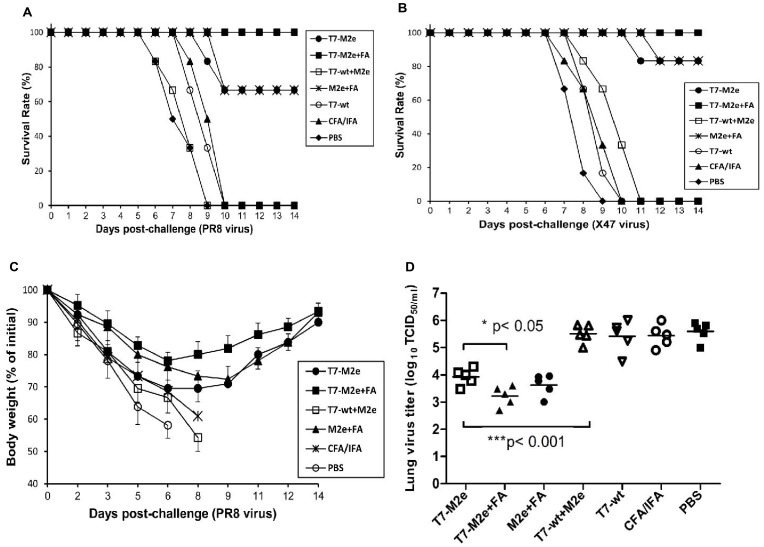
Antiviral effects of T7-M2e nanoparticles. The immunized mice were challenged by 4 LD_50_ of mouse-adapted PR8 (H1N1) or X47 (H3N2) virus 21 days after the second booster dose and monitored for survival and clinical symptoms (body weight loss) for 14 days. T7-M2e nanoparticles without adjuvant significantly protected mice against a lethal infection with both PR8 (A) and X47 (B) viruses as analyzed by Log-rank test. However, addition of CFA/IFA adjuvant resulted in 100% protection of mice. (C) Body weight was monitored for all mice for 14 days post-challenge with PR8 virus. (D) Five days post-challenge, six mice in each group were sacrificed and residual virus titers were measured in lungs as TCID_50_ per ml of each lung homogenate.

Twenty one days after the last injection, mice were challenged intranasally with two mouse-adapted subtypes of influenza A virus: PR8 and X47. T7-M2e nanoparticles, without (T7-M2e group) or with Freund’s adjuvant (T7-M2e+FA group) protected mice against a lethal virus infection (Fig. 5). Immunization with adjuvanted T7-M2e nanoparticles increased the survival rate of mice by 34% and 17% after challenge with PR8 and X47 viruses, respectively, compared to non-adjuvanted immunization. Also, without adjuvant, mice experienced more severe body weight loss, although all animals in this group eventually recovered after challenge (Fig. 5c). It has been reported that immunization with M2e peptide formulated in CFA/IFA induced a high anti-M2e IgG titer and protected mice against a lethal challenge with H3N2 and H1N1 (PR8) influenza virus [45], [46]. However, in our immunization/challenge setting using a similar vaccine formulation we observed only partial protection and no significant difference in survival rate compared to mice receiving T7-M2e nanoparticles in the absence of the adjuvant. All control mice (T7-wt, Freund’s adjuvant and PBS groups) showed excessive weight loss and succumbed to a lethal challenge with both viruses.

### Immunization with T7-M2e Nanoparticles Significantly Reduced Influenza Virus Load in the Lungs

Challenge with PR8 virus of mice that had been immunized with T7-M2e nanoparticles also resulted in a significant reduction of lung virus titers compared to T7-wt, PBS and CFA/IFA control groups (p<0.001) as shown in figure 5. However, addition of Freund’s adjuvant significantly enhanced the antiviral effects of T7-M2e nanoparticles as revealed by significantly lower lung virus titer (p<0.05). Overall, survival rates and reduction of lung virus titers correlated well in our model. No significant reduction was observed in virus titers of mice inoculated with a combination of M2e peptide and T7-wt nanoparticles (T7-wt+M2e group) compared with negative controls (Fig. 5d). Finally, to document protection by the immunization with T7-M2e nanoparticles against a different influenza subtype virus, six mice from each group were challenged with X47 virus, a mouse-adapted H3N2 subtype virus. The mice inoculated with T7-M2e nanoparticles either with or without CFA/IFA survived upon challenge with X47 virus with a survival rate of 100% and 83%, respectively (Fig. 5b). In contrast, all control mice died by day 10–11 after challenge with this H3N2 virus.

## Discussion

A large number of studies have focused on the use of the conserved M2e of influenza A viruses as a candidate vaccine antigen for development of a universal influenza vaccine [7], [47]. Many researchers have been trying to overcome the naturally poor immunogenicity of M2e peptide by coupling it to an appropriate carrier such as VLPs, multiple branched peptides or TLR ligands [28], [29], [30], [32], [48]. In this study, we have exploited a bacteriophage T7-based epitope display system to generate a strong M2e-immunogen. Physical stability and several advantageous biological features of T7 phage nanoparticles including unique multivalency (415 copies per particle), easy and rapid propagation in laboratory strains of *E. coli*, lack of requirement of displayed peptides to be secreted through the bacterial periplasm and remarkable stability of T7 nanoparticles in harsh denaturing conditions [43], encouraged us to exploit this as a platform for an M2e-based universal flu vaccine. Generally, bacteriophage nanoparticles are believed to have no intrinsic tropism towards eukaryotic cells and no phage multiplication in mammalian cells has been detected [49]. Bacteriophage-derived nanoparticles have been extensively investigated as carriers to enhance immunogenicity of various peptides and proteins. This has been achieved either by chemical conjugation or genetic fusion of various epitopes of immunodominant antigens to the surface of icosahedral or filamentous capsids derived from fd [50], [51], [52], lambda [53], [54], [55] and T4 phages [56], [57], [58], [59], [60]. Oral route of administration has also been explored with bacteriophage nanoparticles. For example, it was shown that orally delivered foot-and-mouth disease virus capsid protein displayed on the surface of T4 nanoparticles conferred a 100% protection against challenge with this Picornavirus in mice [61]. T7 phage nanoparticles displaying Ep15 peptide of West Nile virus have been exploited for detection of anti-WNV IgG in patient sera [33] and induction of antibodies by the same nanoparticles carrying the immunodominant region of Hepatitis B virus surface antigen (HBsAg) has been demonstrated in rabbits [34]. In the present study, we investigated whether T7 phage nanoparticles carrying a high density (415 copies per particle) of M2e peptide on the surface as a genetic fusion to 10B capsid protein could induce protective immunity against a lethal challenge with influenza viruses in mice. M2e peptide was stably displayed on the surface of T7 nanoparticles and was accessible to anti-M2e monoclonal antibody 14C2. To exclude innate immunostimulatory effects exerted by bacterial LPS, a purification procedure was adopted from Aida *et al.* with some modifications. We determined the induction of humoral as well as T cell responses directed against M2e by these T7-M2e nanoparticles in the presence or absence of a strong adjuvant (CFA/IFA). T7-Me nanoparticles without any additional adjuvant successfully elicited both IgG_1_/IgG_2a_ and T cell responses in mice after three subcutaneous immunizations. Moreover, a high level of protection (66% and 83% survival rates) was achieved by immunization with T7-M2e nanoparticles against lethal challenge with two different mouse-adapted subtypes of influenza A virus (PR8 and X47 viruses). However, when T7-M2e nanoparticles were emulsified in CFA for priming and IFA for booster injections, significantly higher M2e-specific IgG titers especially of Th_1_-induced IgG_2a_ isotype were obtained and mice were completely protected against challenge with both viruses. Similar results were reported by Denis *et al.* where immunization of mice with M2e-modified Papaya mosaic VLPs in the absence of adjuvant conferred only a partial protection against challenge (50% survival), while co-administration of Alum adjuvant significantly improved the survival rate of mice and increased the level anti-M2e IgG_1_ without a significant influence on IgG_2a_ titers [44]. In other words, Alum improved a Th_2_-oriented response while CFA/IFA adjuvant in our study greatly increased both IgG_2a_ levels in sera and IFN-γ secretion in splenocytes upon re-stimulation with M2e peptide *in vitro*, indicating a Th_1_-dominant response. Even in a setting where free M2e peptide was use in combination with CFA/IFA adjuvant, substantial protection at the level of morbidity and lung virus titers was observed. Mice from this group had significantly higher numbers of IFN-γ secreting splenocytes in response to M2e-peptide re-stimulation compared with T7-M2e immunized mice, even though antibody-titers directed against M2e were comparable between these two groups (compare Fig. 3A and Fig. 4A). This finding may suggest that IFN-γ production by M2e-specific T cells is an important correlate of protection for M2e-based immunity in the mouse model, in addition to M2e-specific humoral immunity. An M2e-specific CD4 T cell response in the BALB/c mouse model has been described following mucosal immunization with M2e-VLPs in adjuvanted with CTA1-DD, a safe and effective cholera toxin derived adjuvant [62]. Generally, mouse IgG_2a_ isotype restrict viral infections better than IgG_1_ Abs [63], [64]. This is further confirmed by the observation that isotype switching of 14C2 anti-M2e monoclonal antibody from IgG_1_ to IgG_2a_ improves its protective efficacy *in vivo* [65], [66]. The exact mechanism(s) by which antibodies induced by M2e vaccination mediates protection against a lethal infection has not yet been completely elucidated. However, NK cells and alveolar macrophages play a crucial role as described by Jegerlehner *et al.* [48] and El Bakkouri *et al*. [15]. IFN-γ secreted by Th_1_ cells promotes B cell isotype switching to IgG_2a_ which strongly mediates opsonization and ADCC by binding via its Fc portion to receptors on a variety of cell types including macrophages and NK cells [16], [17], [18]. Interestingly, T7-M2e nanoparticles without adjuvant elicited a high frequency of M2e-specific IFN-γ-secreting splenocytes (Fig. 4) which favored production of IgG_2a_, in amounts comparable to those from mice immunized with M2e peptide combined with CFA/IFA adjuvant (Figure 3). Furthermore, most of the mice that had received T7-M2e nanoparticles survived upon lethal challenge with two different subtypes of mouse-adapted influenza viruses (PR8 and X47) indicating a desirable cross-protectivity of the described platform. No significant difference was observed in survival rates of T7-M2e and M2e+FA groups demonstrating high immunogenicity of M2e peptide when displayed densely on the surface of T7 nanoparticles (Fig. 5). By contrast, when simply mixed with T7-wt nanoparticles, M2e peptide only triggered a minimal response against the M2e peptide, not strong enough to protect mice against a lethal virus infection or clinical symptoms (body weight loss). In accordance, Denis *et al.* previously reported that a mixture of PapMV VLPs with free M2e peptide failed to induce antibodies against M2e and to protect mice against lethal challenge [44]. Our observation further confirms the key role of particulate delivery and multivalent arrangement of M2e peptide on the surface of nanoparticles in triggering a strong activation signal via cross-linking of B cell receptors and subsequent antibody production [22]. Direct comparison in an experimental efficacy study of the T7-M2e nanoparticle vaccine described here with e.g. M2e-MAP, M2e-HBcore or M2e-QBeta fusion constructs would be required to rank the protective potential of different multivalent M2e epitope display systems. We believe that the prime benefit of the T7-M2e nanoparticle vaccine candidate is primarily an economical one: the technology to produce and isolate T7-based particles requires a safe prokaryotic cell system and technically straightforward physical separations steps to obtain.

No significant increase in IgG_1_ isotype levels were detected in the mice receiving T7-M2e nanoparticles compared to those inoculated with a T7-M2e/CFA/IFA emulsion. Similarly, a multiple antigenic peptide (MAP) platform carrying multiple copies of M2e peptide elicited higher IgG titers when administered with CFA/IFA than with Alum compared to adjuvant-less administration. Also, lower virus titers were detected in lungs of mice receiving MAP-CFA/IFA [26], [27]. In contrast, only background level of antibody responses and IFN-γ-positive spots were detected in the mice receiving CFA/IFA adjuvant alone or T7-wt nanoparticles. Quantification of residual influenza viruses in lungs showed that when challenged with virus, the mice inoculated with T7-M2e nanoparticles were able to effectively clear virus from their lungs comparable to those injected with M2e peptide plus CFA/IFA (Fig. 5d). However, addition of CFA/IFA to T7-M2e nanoparticles conferred a superior protection against a lethal infection with both PR8 and X47 viruses and significantly inhibited virus propagation in lungs. These results correlated well with M2e-specific IgG2a titers in mice sera (Fig. 3). Naturally occurring bacteriophages are able to induce humoral immunity as phage-neutralizing antibodies have been detected in human and animal sera probably reflecting frequent natural contact of humans/animals with various phage species [17], [63]. So we decided to measure anti-T7 nanoparticles IgG in sera in addition to M2e-specific Abs. Induction of anti-M2e IgG response was not inhibited in mice immunized three times with T7-M2e nanoparticles either with or without adjuvant, because high anti-M2e IgG titers were detected in sera despite high levels of anti-T7 carrier IgG Abs. This phenomenon known as carrier-induced epitopic suppression (CIES) was observed with Qβ VLPs particularly if VLPs chemically conjugated with low peptide densities were used [67]. Furthermore, the researchers reported that the observed suppression could be reduced or overcome by conjugating the peptide to the VLP at high density, by repeated injections and increasing the dose of the vaccine. Another study by De Filette *et al.* showed that vaccination with M2e-HBc VLPs can induce protective anti-M2e antibodies even in the presence of pre-existing anti-HBc carrier antibodies [68]. In the present study, we injected mice three times with a relatively high dose of T7-M2e nanoparticles (10^9^ pfu/mouse) carrying a high copy of M2e peptide per particle which probably inhibited the epitope suppression by anti-T7 IgG. The data presented here suggest that pre-existing anti-T7 phage antibodies in human population would not interfere with the rise of anti-M2e peptide antibodies in booster administrations or compromise the efficacy of the induced protection. However, the proof will have to come from clinical trials involving individuals who are positive for anti-T7 phage IgG.

In conclusion, our findings suggest that T7-phage nanoparticles carrying M2e peptide can be considered as a promising strategy to induce protective antibodies against multiple subtypes of influenza A viruses. However, M2e does display sequence variation in circulating human influenza viruses. With few exceptions, this sequence variation is confined to the membrane proximal part of M2e (amino acid residues 10–23). For example, there are 4 amino acid differences between M2e as used here in T7-M2e nanoparticles (Fig. 1) and A/California/04/09 virus. Such sequence variation likely affects protection induced by an M2e-based vaccine strategy in case of an insufficient match between M2e and the challenge virus. It has been reported that a close match between the M2e sequence and the challenge virus improves protection afforded by M2e-specific antibodies compared to an incomplete match [69], [70]. Although still experimental, in the worst-case scenario of the pandemic spread of lethal influenza viruses, a T7 phage-based universal vaccine could be co-administered to broaden immunogenicity of currently used seasonal influenza vaccines as evaluated by Wu *et al*. using a combination of M2e peptide and split influenza vaccine [71]. However, more pre-clinical evaluation of these nanoparticles is required in larger animals and humans. Finally, it is important to point out that Freund’s adjuvant is a very strong inflammatory adjuvant, and is not licensed for human use. Freund’s adjuvant is still regularly used in experimental studies in mice to obtain proof-of-concept for novel influenza vaccine candidates [72]. Currently, aluminum salts (Alum) are the only human vaccine adjuvants licensed by the Food and Drug Administration (FDA), however in recent years, oil in water emulsions such as MF59 and AS03 have been licensed for adjuvanted influenza vaccines in Europe [73]. While Alum is safe, it is a mild Th_2_ adjuvant that can effectively enhance IgG_1_ antibody responses, but it is rarely associated with Th_1_ type immune responses [74]. Hence, a myriad of researches have been conducted to develop a safe and efficacious adjuvant capable of boosting cellular plus humoral immunity [75]. Such adjuvants would be expected to have the potential to both intensify the immune response and also to reduce the dose of vaccine antigen needed per vaccination or the number of injections.
